# Cerebrospinal fluid and peripheral blood proteomics in Traumatic Spinal Cord Injury: A prospective pilot study

**DOI:** 10.1016/j.bas.2022.100906

**Published:** 2022-06-15

**Authors:** Thea Overgaard Wichmann, Helge Kasch, Stig Dyrskog, Kristian Høy, Bjarne Kuno Møller, Jan Krog, Hans Jürgen Hoffmann, Claus Vinter Bødker Hviid, Mikkel Mylius Rasmussen

**Affiliations:** a Dept. Neurosurgery, Cense-Spine, Aarhus University Hospital, Aarhus, Denmark; b Dept. Neurology, Viborg Regional Hospital, Toldbodgade 12, 8800, Viborg, Denmark; c Dept. of Clinical Medicine, Aarhus University, Palle Juul-Jensens Boulevard 82, 8200, Aarhus N, Denmark; d Dept. Intensive Care, Aarhus University Hospital, Palle Juul-Jensens Boulevard 99, 8200, Aarhus N, Denmark; e Dept. Orthopaedic Surgery – Spine Section, Aarhus University Hospital, Palle Juul-Jensens Boulevard 99, 8200, Aarhus N, Denmark; f Dept. of Clinical Immunology, Aarhus University Hospital, Palle Juul-Jensens Boulevard 99, 8200, Aarhus N, Denmark; g Dept. Anaesthesiology, Aarhus University Hospital, Palle Juul-Jensens Boulevard 99, 8200, Aarhus N, Denmark; h Dept. Respiratory Diseases and Allergy, Aarhus University Hospital, Palle Juul-Jensens Boulevard 93, 8200, Aarhus N, Denmark; i Dept. Clinical Biochemistry, Aarhus University Hospital, Palle Juul-Jensens Boulevard 99, 8200, Aarhus N, Denmark

**Keywords:** Traumatic spinal cord injury, Pathophysiology, Inflammation, Proteomics

## Abstract

• Proteomics enable profiling of inflammatory responses after spinal cord injury.
• Proteins are differentially expressed over time.
• Proteins are differentially expressed in cerebrospinal fluid and peripheral blood.
• A poor relationship exists between protein expression and neurological outcome.

## 1 Introduction

Traumatic spinal cord injury (TSCI) is a heterogenic condition with a complex pathophysiology (Hachem and Fehlings, 2021; Alizadeh et al., 2019). The complexity of the pathophysiology is reflected by the multitude of secondary processes that arise and evolve after the primary injury (Hachem and Fehlings, 2021; Alizadeh et al., 2019). The inflammatory response is a central secondary pathophysiological process (Alizadeh et al., 2019; Anwar et al., 2016; Liu et al., 2021). Although intensive research has unveiled several aspects of this response after TSCI, most of our knowledge of the inflammatory response derives from animal studies (Skinnider et al., 2021). Therefore, human studies are needed to verify the pathophysiological findings of these studies. Proteomics enable broad profiling of inflammatory biomarkers and is therefore a powerfull tool to assess the inflammatory response and thereby, advance our knowledge of the pathophysiology. Despite growing interest in the field of proteomics (Skinnider et al., 2021; Dalkilic et al., 2018; Kwon et al., 2010; Pouw et al., 2014; Kwon et al., 2017; Streijger et al., 2017; Fern á ndez et al., 2020; Kuhle et al., 2015; Huang et al., 2014; Capirossi et al., 2020; Sengupta et al., 2014), human studies of cerebrospinal fluid (CSF) and peripheral blood (PB) collected throughout the acute, subacute, and chronic phase of injury are scarce with only two proteomic studies of the acute phase of TSCI (Skinnider et al., 2021; Kwon et al., 2010). It is, however, important to study CSF and PB over time in TSCI to gain knowledge of proteomic dynamics locally and systemically. Furthermore, it is important to study if proteomic dynamics reflect clinical neurological outcome.

This study therefore aimed to 1) perform a targeted proteomic analysis using proximity extension assay (PEA) technologies to describe local and systemic inflammatory responses throughout the acute, subacute, and chronic phase of TSCI, and to 2) explore the relationship between inflammatory protein expression and clinical neurological outcome.

## 2 Materials and methods

### 2.1 Patient population

This study is based on data from our prospective pilot study exploring inflammatory responses and biochemical biomarkers in TSCI patients (ClinicalTrials.gov, clinical trial reg. no: NCT03505463). Approval was obtained by the Central Denmark Region Committees on Health Research Ethics (1-10-72-382-17) and the Danish Data Protection Agency (1-16-02-754-17) and conformed to the Declaration of Helsinki. We included TSCI patients at the Department of Neurosurgery at Aarhus University Hospital, Denmark, between 2018 and 2020. We further included a reference group of patients without spinal cord injury (Non-TSCI patients) scheduled for elective hip surgery in 2020 ​at the Department of Orthopaedic Surgery at Aarhus University Hospital, Denmark. Patients provided written consent before inclusion. Inclusion and exclusion criteria for TSCI patients are presented in Table 1. Given the pilot design, no sample size calculation was performed. The number of patients in the reference group was decided based on the number of TSCI patients.

**Table 1:** Inclusion and exclusion criteria for study participants with traumatic spinal cord injury.

Inclusion criteria	Traumatic spinal cord injury bony level C1/L1
	Glasgow Coma Scale 15 upon admission
	American Spinal Injury Association Impairment Scale score A-D upon admission
	≥18 years of age upon admission
Exclusion criteria	Prior spine surgery at level of injury
	Major co-injuries, including traumatic brain injury
	Major co-morbidities, including immunological disease and other neurological disease
	Immune suppression treatment
	Penetrating spinal cord injury
	>72 ​h from injury until sample collection
	Substance abuse
	Mental disease preventing compliance with study procedure

### 2.2 Clinical data and sample collection

Clinical data on age, sex, mechanism of injury, level of injury and treatment of injury were gathered from medical records. Clinical neurological outcome was assessed at three time-points by trained physicians or physiotherapists according to the American Spinal Injury Association (ASIA) Impairment Scale (AIS) (Roberts et al., 2017). Samples of CSF and PB were collected at the same three time-points from TSCI patients and at one-time-point from non-TSCI patients. TSCI patients had CSF collected by lumbar puncture. The first lumbar puncture was performed prior to or immediately after surgery with the patient log-rolled to the side and the spinal column kept in a neutral position. A 22- or 25-gauge spinal needle was inserted in the L2-3, L3-4 or L4-5 interspace and 4–5 ​ml CSF was aspirated into a glass tube. Non-TSCI patients had CSF collected in conjunction with spinal anesthesia before administration of anesthetic agents. A 25-gauge spinal needle was inserted in the L2-3, L3-4 or L4-5 interspace and 3 ​ml CSF were aspirated into a glass tube. PB was collected from an arterial catheter or by venepuncture into a 6 ​mL ethylenediaminetetraacetic (EDTA)-containing tube (BD Vacutainer®, Becton Dickison and Company, Franklin Lakes, NJ, USA). Details on the time from collection to processing is presented in the supplentary.

### 2.3 Proteomic analysis

Measurement of 92 cytokines, chemokines, and other inflammatory proteins was carried out by BioXpedia A/S using the Olink Target 96 Inflammation panel (Olink Proteomics AB, Uppsala, Sweden) according to the manufacturer's instructions (Assarsson et al., 2014). A list of protein names, sources and functions is provided by Dyhrfort et al. (2019). Protein concentrations is provided as normalized protein expression (NPX) values. NPX values are arbitrary values on a log_2_-scale, i.e., an increase of 1 NPX represents a two-fold increase of protein concentration. Every NPX value has its own lower limit of detection value. A total of 11 CSF proteins (Glial cell line-derived neurotrophic factor (GDNF), interleukin-2 receptor subunit beta (IL-2RB), IL-2, Signaling lymphocytic activation molecule (SLAMF1), IL-22 receptor subunit alpha-1 (IL-22RA1), Beta-nerve growth factor (Beta-NGF), TNF-related activation-induced cytokine (TRANCE), IL-24, IL-13, Neurturin (NRTN), IL-5) and 4 ​PB proteins (IL-2RB, IL-1 alpha, Beta-NGF, IL-13) were omitted from further analyses as >90% of the measurements were below lower limit of detection. Proteins were grouped into categories of primary effects: induction of inflammation, recruitment of granulocytes, monocytes and macrophages, and other proteins.

### 2.4 Statistical analyses

For descriptive analyses, continuous variables are reported as mean with range, or median with interquartile range (IQR) depending on the data distribution. Unpaired continuous variables were compared by t-tests or Mann-Whitney U-tests. Categorical variables are reported as absolute numbers (n) with percentages (%). Categorical variables were compared by Chi squared tests or the Fisher's exact tests. For protein analyses, the following associations were tested using unpaired t-tests or paired t-tests: I) protein expression between TSCI and non-TSCI patients; II) protein expression in TSCI patients across the three time-points; III) protein expression and acute AIS grades; and IV) protein expression and AIS grade conversion. AIS grades were modeled as an ordinal variable and AIS grade conversion was modeled as a binary variable. The Shapiro-Wilk test was used to test data normality. If one of the groups were not normally distributed a Wilcoxon rank sum test or signed rank test were used. The fold change in protein expression was calculated on a linear scale as the geometric mean of the first group divided with the geometric mean of the second group. P-values were adjusted for multiple testing using the Benjamini-Hochberg method. The Benjamini-Hochberg method decreases the false discovery rate i.e., controls for small p-values happen by chance. thereby avoiding type 1 errors. Both unadjusted p values and adjusted p values are reported. Statistical significance was defined as p ​≤ ​.05. All statistical analyses were performed using R software version 3.6.1 by BioXpedia A/S.

## 3 Results

### 3.1 Characteristics of study population

For details on characteristics of study population see Table 2. Fifteen TSCI patients and fifteen non-TSCI patients were included. TSCI patients and non-TSCI patients were not statistically significant different regarding sex and age. Median follow-up was day 0 (IQR: 1), day 9 (IQR: 2) and day 148 (IQR: 49) after TSCI. The follow-up are reffered to as the acute, subacute, and chronic phase of TSCI. Four TSCI patients withdrew their consent after the acute phase.

**Table 2:** Demographics of the study population.

	TSCI patients	Non-TSCI patients^a^
Count, n	15	15
Sex, n (%)		
Women	4 (27%)	7 (47%)
Men	11 (73%)	8 (53%)
Age, mean year (range)	50 (21–75)	51 (18–82)
Follow-up, median days (IQR)		
Acute	0 (1)	
Subacute	9 (2)	
Chronic	148 (49)	
Mechanism of injury, n (%)		
Fall	7 (47%)	
Traffic accident	7 (47%)	
Sports	1 (7%)	
Level of injury, n (%)		
Cervical	11 (73%)	
Thoracic	4 (27%)	
Lumbar	0 (0%)	
AIS grade, n (%)		
A	6 (40%)	
B	2 (13%)	
C	4 (27%)	
D	3 (20%)	
AIS grade conversion, n (%)^b^		
Yes	4 (36%)	
No	7 (64%)	

AIS: American Spinal Cord Association Impairment Scale; TSCI: Traumatic Spinal Cord Injury.

a Non-TSCI refers to the reference group of patients without spinal cord injury.

b Only eleven TSCI patients had complete follow-up.

### 3.2 CSF protein expression

In the acute phase, 37 proteins were significantly changed in TSCI patients compared with non-TSCI patients. Proteins were predominantly upregulated and involved in chemotaxis of granulocytes, monocytes, macrophages, and lymphocytes; however, proteins involved in degradation of extracellular matrix and apoptosis were also upregulated (Table 3). Comparing TSCI patients in the subacute phase to non-TSCI patients, a total of 24 proteins displayed significant differences in expression. The protein composition resembled that of the acute phase; however, fewer proteins involved in chemotaxis of neutrophils were upregulated. In the chronic phase, a total of 12 proteins displayed significant differences in expression in TSCI patients compared to non-TSCI patients. Proteins were mainly involved in chemotaxis of monocytes, macrophages, and lymphocytes. Only few proteins (CCL3, CCL23, OSM, MCP-1, TNF, CXCL10, MMP-10, and IL-18) were regulated through all phases while the majority were distinct between the three phases of the injury. All differences were statistically significant after correction for multiple tests. A complete list and volcano plots of protein expression across injury phases and compartments are presented in the supplementary.

**Table 3:** Proteins being significantly up- or downregulated in cerebrospinal fluid after correction for multiple testing.

Key protein function		D0 vs non-TSCI			D9 vs non-TSCI			D148 vs non-TSCI		
		Log_2_FC	P_Adj_ value	Regulation	Log_2_FC	P_Adj_ value	Regulation	Log_2_FC	P_Adj_ value	Regulation
Inflammation										
IL-1alpha	Initiation of inflammation.	0.82	6.8E-03*							
IL-6	Pro-inflammatory cytokine	6.28	2.1E-05*		3.13	1.8E-03*				
IL-10	Anti-inflammatory cytokine	2.19	6.1E-05*							
IL-10RB	Required for IL-10-induced signal transduction				0.93	1.4E-02*				
IL-18	Pro-inflammatory cytokine	1.91	6.6E-05*		1.10	6.9E-03*		0.82	3.2E-02*	
IL-20RB	Involved in epidermal function				−0.26	1.6E-02*				
CCL20	Pro-inflammatory cytokine	4.05	9.5E-04*							
CD244	Modulation of leukocyte activation	0.46	4.5E-02*							
OSM	Pro- and anti-inflammatory cytokine	3.48	3.3E-05*		1.54	1.9E-02*		0.38	3.2E-02*	
OPG	Transcriptional regulation in inflammation	0.99	8.8E-03*							
Granulocytes										
IL-8	Pro-inflammatory cytokine	4.29	3.4E-06*		1.61	1.8E-03*				
CCL3	Recruitment and activation of polymorphonuclear leukocytes	2.16	1.5E-03*		1.02	2.9E-02*		1.23	2.3E-03*	
CCL4^§^	Chemotactic for neutrophils, monocytes, T cells, B cells	2.17	6.4E-04*							
CCL11	Promotion of eosinophil accumulation	0.77	2.7E-02*							
CXCL1	Chemotactic for neutrophils	3.93	2.7E-04*							
CXCL5	Chemotactic for neutrophils	1.33	1.2E-02*							
CXCL6	Chemotactic for neutrophils	2.34	2.5E-03*		1.30	1.2E-02*				
MCP-1^§^	Chemotactic for basophils and monocytes*	2.02	1.2E-05*		0.98	5.2E-04*		1.09	3.0E-03*	
MCP-2^§^	Chemotactic for basophils, eosinophils, mast cells, monocytes, T cells, NK cells	1.89	3.7E-05*		1.95	1.1E-02*				
MCP-4^§^	Chemotactic for basophils, eosinophils, monocytes, T cells*	1.03	1.9E-02*		1.53	3.5E-02*				
Monocytes and macrophages										
CCL4^§^	Chemotactic for neutrophils, monocytes, T cells, B cells	2.17	6.4E-04*							
CCL23^§^	Chemotactic for monocytes and resting T cells	1.14	1.1E-03*		1.60	1.4E-03*		1.05	6.0E-03*	
CXCL10^§^	Chemotactic for monocytes, T cells, NK cells, dendritic cells	1.25	5.6E-03*		2.04	1.3E-03*		1.32	2.4E-02*	
MCP-3	Chemotactic for monocytes	2.66	9.7E-04*		2.00	5.3E-05*				
CSF-1	Induction of monocyte and macrophage development	0.70	1.1E-02*							
Lymphocytes										
IL-7	Important for T and B cell development				0.53	2.3E-02*				
CCL28	Chemotactic for T and B cells	0.43	1.5E-02*		0.34	4.4E-02*				
CXCL9	Chemotactic for T cells				1.73	1.4E-02*		1.32	2.8E-02*	
CXCL11	Chemotactic for T cells	0.96	3.7E-02*		1.72	2.8E-02*				
TSLP^§^	Maturation of T cells and activation of JAK/STAT signaling pathways	0.75	7.3E-03*							
TNFRSF9	Active during T cell activation	0.66	3.5E-02*		0.82	4.6E-02*				
CASP-8^§^	Involved in apoptosis and activation of T cells, B cells, NK cells, macrophages	1.44	2.0E-03*							
Other proteins										
X4E-BP1	Regulation of protein translation	2.01	1.1E-03*		1.65	1.7E-02*				
MMP-1	Degradation of extracellular matrix	2.12	4.8E-03*							
MMP-10	Degradation of extracellular matrix	1.44	1.5E-02*		1.13	7.3E-04*		0.94	3.5E-02*	
STAMPBP	Regulation of endocytosis and cell growth	1.52	4.1E-04*		0.67	4.0E-02*				
LIF	Activation of JAK/STAT and MAPK signaling pathways	5.46	3.4E-06*		2.23	1.1E-03*				
ST1A1	Part of metabolism	0.93	2.4E-02*							
AXIN1	Regulation of Wnt signaling pathways	0.63	7.3E-03*							
TNF	Activation of NF-kappaB, MAPK and apoptosis pathways	0.98	1.4E-03*		0.84	6.0E-04*		0.53	1.4E-02*	
FGF-21	Stimulation of glucose uptake	1.32	2.4E-02*							
CDCP1	Negative regulation of cell adhesion							0.53	4.7E-02*	
TWEAK	Activation of apoptosis	−0.79	7.3E-03*							

Log_2_ fold change (log_2_FC) and p values (adjusted using the Benjamini-Hochberg method) for the comparison of non-TSCI patients and TSCI patients. Non-TSCI refers to the reference group of patients without spinal cord injury. A positive log_2_FC value indicates upregulated protein expression in TSCI patients, whereas a negative log_2_FC value indicates downregulated protein expression. §Protein related to more than one group. *Statistical significance was set at p ≤ .05.

### 3.3 PB protein expression

Comparing TSCI patients in the acute phase to non-TSCI patients, a total of 17 proteins were differentially expressed. These proteins belonged to multiple functional groups (Table 4). In the subacute phase, a total of 28 proteins displayed significant differences in expression in TSCI patients compared to non-TSCI patients. Compared to the acute phase, more proteins in the subacute phase were cell specific and predominately involved in monocyte, macrophage, and lymphocyte responses (Table 4). In the chronic phase, only 4 pro-inflammatory proteins (EN-RANGE, FGF-19, TNF, CXCL11) displayed significant differences between TSCI patients compared with non-TSCI patients (Table 4). Proteins were upregulated and involved in inflammation and apoptosis. Only EN-RANGE was shared across the three phases. All differences were statistically significant after correction for multiple tests.

**Table 4:** Proteins being significantly up- or downregulated in peripheral blood after correction for multiple testing.

Key protein function			D0 vs non-TSCI			D9 vs non-TSCI			D148 vs non-TSCI			
			Log_2_FC	P_Adj_ value	Regulation	Log_2_FC	P_Adj_ value	Regulation	Log_2_FC		P_Adj_ value	Regulation
Inflammation												
IL-6		Pro-inflammatory cytokine	2.59	7.9E-04∗		1.65	8.6E-03∗					
IL-10		Anti-inflammatory cytokine	1.95	1.5E-03∗		0.96	9.6E-04∗					
IL-10RB		Required for IL-10-induced signal transduction	−0.40	2.5E-02∗								
IL-17A		Pro-inflammatory cytokine				1.61	1.4E-03∗					
IL-18R1		Important for IL-18 signal transduction				0.94	4.6E-03∗					
IL-20RA		Involved in epidermal function	0.28	9.8E-03∗		0.39	2.1E-02∗					
IL-24		Pro-inflammatory cytokine	0.49	4.6E-02∗								
TNFB		Mediates inflammation	−0.59	1.7E-02∗		0.54	4.6E-02∗					
SCF		Pro-inflammatory cytokine	−0.43	3.2E-02∗		−1.11	4.8E-03∗					
EN-RANGE		Calcium-binding pro-inflammatory protein	1.08	1.3E-04∗		2.87	1.6E-05∗		1.09		6.0E-03∗	
FGF-19		Involved in immune responses				2.03	3.4E-05∗		1.80		8.5E-06	
Granulocytes												
IL-8		Pro-inflammatory cytokine	1.29	7.3E-03∗		1.44	2.9E-02∗					
CCL3		Recruitment and activation of polymorphonuclear leukocytes				1.17	7.7E-03∗					
CCL4§		Chemotactic for neutrophils, monocytes, T cells and B cells				0.83	1.5E-02∗					
CCL11		Promotion of eosinophil accumulation	−0.55	4.6E-02∗								
Monocytes and macrophages												
CCL23§		Chemotactic for monocytes and resting T cells	0.69	6.1E-03∗								
CCL25		Chemotactic for macrophages and dendritic cells	−0.80	2.9E-02∗								
MCP-3		Chemotactic for monocytes	0.89	7.3E-03∗		1.24	1.9E-03∗					
CSF-1		Induction of monocyte and macrophage development				0.57	3.6E-05∗					
CXCL11§		Chemotactic for monocytes, T cells, NK cells and dendritic cells				2.05	2.9E-02∗		1.24		4.7E-02∗	
TNFSF14§		Involved in T cell homing and induction of MMP in macrophages				1.42	8.9E-03∗					
Lymphocytes												
CCL19	Chemotactic for T cells and B cells					0.81	2.0E-02∗					
IL-2	Stimulates T cells proliferation					0.26	1.7E-02∗					
TRANCE	Regulation of T cell responses		−1.23	2.3E-04∗		−0.93	7.1E-03∗					
Other proteins												
MMP-1	Degradation of extracellular matrix					1.40	1.1E-02∗					
MMP-10	Degradation of extracellular matrix					0.76	3.6E-02∗					
LIF	Activation of JAK/STAT and MAPK signaling pathways					0.61	2.1E-02∗					
TNF	Activation of NF-kappaB, MAPK and apoptosis pathways					0.87	1.3E-03∗		0.45	3.7E-02∗		
FGF-21	Stimulation of glucose uptake					−1.69	1.8E-02∗					
TWEAK	Activation of apoptosis		−0.48	4.6E-02∗		−0.76	1.3E-03∗					
TRAIL	Activation of apoptosis		−0.46	1.0E-02∗								
Fit3L	Involved in dendritic cell development		−084	5.0E-04∗								
NT-3	Promotion of survival and differentiation of neurons					−0.27	3.7E-02∗					
DNER	Activation of NOTCH1 pathway					−0.51	3.7E-02∗					
VEGFA§	Induction of endothelial cell proliferation, promotion of cell migration, inhibition of apoptosis, induction of blood vessel permeabilization, stimulation of monocytes and macrophage migration					0.87	3.6E-02∗					

Log_2_ fold change (log_2_FC) and p values (adjusted using the Benjamini-Hochberg method) for the comparison of non-TSCI patients and TSCI patients. Non-TSCI refers to the reference group of patients without spinal cord injury. A positive log_2_FC value indicates upregulated protein expression in TSCI patients, whereas a negative log_2_FC value indicates downregulated protein expression. §Protein related to more than one group. ∗Statistical significance was set at p ​≤ ​.05.

### 3.4 Protein expression across compartments

The protein expression profiles differed profoundly between CSF and PB; however, there was an overlap between some differential expressed proteins in CSF and PB e.g., IL-6, IL-8, IL-10, CCL23, MCP-3, TWEAK, and CCL11 (Table 3, Table 4). Differentially expressed CSF proteins were largely upregulated with highest number of regulated proteins observed in the acute phase. In contrast, differential expressed PB proteins were both up- and downregulated with highest number of regulated proteins observed in the subacute phase.

### 3.5 Protein expression and neurological outcome

We assessed the association between protein expression in the acute phase and the acute AIS grade. As only two TSCI patients had AIS grade B injuries, these patients were omitted from analysis. In CSF, some proteins revealed statistically significant differences in expression among AIS grade A, C, and D; however, after multiple testing correction, only VEGFA (p ​= ​.026) was differently expressed in AIS A compared to AIS C, while LIF (p ​= ​.019) and CLL3 (p ​= ​.036) was differently expressed in AIS A compared to AIS D. In PB, few proteins revealed statistically significant differential expression among AIS grade A, C, and D. After multiple testing correction, only MMP-10 (p ​= ​.038) and LIF-R (p ​= ​.041) were differently expressed in AIS A compared to AIS D, and CCL20 (p ​= ​.013) in AIS C compared to AIS D.

We further assessed the association between protein expression and neurological recovery as quantified by AIS grade conversion. Again, we focused on CSF and PB samples collected in the acute phase. Although some proteins in CSF and PB were significantly differential expressed between TSCI patients experiencing conversion and not experiencing conversion, the analysis revealed only three specific proteins whose association was statistically significant after multiple testing correction. GDNF (p ​= ​.017) was upregulated in PB of TSCI patients experiencing AIS grade conversion, while SIRT2 (p ​= ​.031) and IL1-alpha (p ​= ​.039) were downregulated in CSF of TSCI patients not experiencing AIS grade conversion.

## 4 Discussion

Our findings demonstrate that profound changes occur in the inflammatory proteome after human TSCI. Proteins had distinct up- and downregulation patterns in the three phases after injury, and the proteins exhibited distinct changes in the two compartments. Only few proteins were significantly associated with injury severity and neurological recovery and none of these proteins were shared between the two compartments.

### 4.1 Distinct proteomic profiles across phases

The proteomic changes differed across the three phases with only few proteins being shared. In CSF, the greatest number of upregulated proteins were observed in the acute phase after the injury. In line with previous cellular findings in CSF (Zrzavy et al., 2021), chemoattractants of neutrophils prevailed in the acute phase, whereas chemoattractants of monocytes, macrophages and lymphocytes prevailed in the subacute and chronic phase. Thus, consistently with previous studies, neutrophils are the predominant leukocytes in the acute inflammatory response to TSCI (Zrzavy et al., 2021; Fleming et al., 2006).

In PB, the greatest number of regulated proteins were observed in the subacute phase, indicating that systemic activation of the inflammatory response occur at later stages after the injury than in the CSF. These observations are in line with the results presented by others who also suggest that the systemic inflammatory response develops over longer time than the local inflammatory response (Skinnider et al., 2021). Consistent with prior studies (Zrzavy et al., 2021; Fleming et al., 2006; Yang et al., 2004; Schwab et al., 2014), our results also suggest the inflammatory response to resolve over time after the injury, though with some degree of persisting inflammatory activity in the chronic phase. The chronic nature of the inflammatory response appears to be more pronounced locally in CSF than systemically in PB. These results suggest that the clearance mechanisms within the spinal cord should be addressed through further research as these mechanisms might explain why the inflammatory response persist after TSCI. Thus, the prolonged local inflammatory response might be a result of ineffectively protein clearance and disturbed homeostasis in the microenvironment. This might consequently impair neurological recovery. Similar considerations have been addressed in relation to other neurological diseases marked by an inflammatory response (Mogensen et al., 2021; Filiano et al., 2017).

### 4.2 Distinct proteomic changes across compartments

The proteomic changes were profound in each of the investigated compartments; however, only few of the assayed proteins were shared. This finding is consistent with prior studies of human TSCI (Skinnider et al., 2021; Kwon et al., 2010), and suggest that tissue specific inflammatory responses arise after TSCI. The CSF, regulated proteins were predominately involved in chemotaxis of granulocytes, monocytes, macrophages, and lymphocytes. Elevations of these proteins in the CSF is likely to reflect the recruitmet of immune cells to the injury site in the spinal cord. Proteins involved in degradation of extracellular matrix e.g., MMP-1 and apoptosis e.g., CASP-8 were also upregulated throughout the three phases in the CSF. A study of traumatic brain injuries suggest MMP-1 and perhaps MMP-10 to be involved in breakdown of BBB and edema formation (Dyhrfort et al., 2019); however, this warrants further investigation. Interestingly, the regulated proteins in PB had multiple functions related to induction of inflammation and other signaling pathways, and thus different to the proteins in CSF. Yet, the structure of the blood-spinal cord barrier is thought to be disrupted after TSCI, leading to spillover of components between the two compartments (Jin et al., 2021). Considering our findings of distinct proteomic profiles and distinct regulation patterns of some proteins, it seems questionable that spillover of proteins occurs through a disrupted blood-spinal cord-barrier. Together these findings highlight the need for careful biomaterial selection when designing future TSCI studies. Furthermore, these findings highlight the need for addressing whether the blood-spinal cord-barrier become disrupted or permeable after TSCI, and the optimal measure of blood-spinal cord-barrier disruption and permeability should also be addressed.

### 4.3 Poor relationship between inflammatory proteins and outcome

We assessed the relationship between protein expression and acute AIS grades to explore if protein expression was related to injury severity. Only few proteins were statistically significant differential expressed among AIS grade A, C, and D; however, none of the proteins were shared between the two compartments. We furthermore assessed the relationship between protein expression and AIS grade conversion to explore if protein expression was related to neurological recovery. Only few TSCI patients experienced improvement in AIS grade and only few proteins had a statistically significant association with AIS grade improvement. Again, none of the proteins were shared between the two compartments. A large-scale study conducting a targeted proteomic analysis of a wide variety of CSF and PB proteins report similar results (Skinnider et al., 2021). These findings might be attributed to the insensitivity of AIS to capture the heterogeneity of TSCI, the differences in injury level, the differences in trauma impact and the small number of patients. Furthermore, these findings might be attributed to the presence of inflammatory proteins throughout the body, the complexity of the immune system, and the variability in immune systems. Future studies should therefore focus on proteins more specific for central nervous system injury e.g., glial fibrillary acidic protein. This would increase the clinical applicability of the data.

### 4.4 Strengths and limitations

Our study is the first prospective study using targeted proteomic analysis in CSF and PB collected in the acute, subacute, and chronic phase of TSCI. As such, it provides valuable insight into how inflammatory responses evolve locally and systemically after human TSCI, and how inflammatory responses relate to clinical neurological outcome. Yet, the results of our pilot study must be interpreted cautiously as the small sample size and the hetereogenity of the study population reduce the statistical power. The sample size was limited by the relatively rare nature of the condition and the strict inclusion criteria, and this led to a heterogenous study population, making it difficult to explore the relationship between protein expression and outcome. Due to the small sample size, we may not have the power to make any definitive conclusions, notably protein expression and outcome; however, our study is rare and allows for a general description of the inflammatory response in different compartments along time after the injury. As enzyme-linked immunosorbent assay (ELISA) technologies are less sensitive for proteins that exhibit relatively low detectability, we utilized PEA technologies in the present study as this technique is extremely specific and sensitive. The PEA technology furthermore enables measurement of greater numbers of proteins than the ELISA technology. Still, utilizing a targeted approach does not give the opportunity to explore proteins beyond the panel (Assarsson et al., 2014; Lundberg et al., 2011).

## 5 Conclusion

This study points towards distinct inflammatory proteomic changes arise and evolve locally and systemically after TSCI, and that a poor relationship exists between protein expression and clinical neurological outcome.

## Funding

This work was supported by Lundbeckfonden; Aase og Ejnar Danielsens Fond; Grosserer L.F. Foghts Fond; Dagmar Marshalls Fond; A.P. Møller Fonden; Grosserer A.V. Lykfeldt og Hustrus Legat; Jascha Fonden; and UlykkesPatientForeningen.

## Author contributions

Conceptualization, TOW, HK, SD, CVBH, HJH, and MMR; Methodology, TOW, HK, JK, SD, CVBH, HJH, and MMR; Formal analysis, TOW, BKM, JK, CVBH, HJH, and MMR; Data curation, TOW, HK, SD, KH, and MMR; Writing—original draft preparation, TOW; Writing—review and editing, all authors; Visualization, TOW; Funding acquisition, TOW. All authors have read and agreed to the published version of the manuscript.

## Disclosures

The authors declare that they have no conflicts of interest.

## Publication

The material has not been published or submitted for publication elsewhere.

## Declaration of competing interest

The authors declare that they have no known competing financial interests or personal relationships that could have appeared to influence the work reported in this paper.

## References

[bib1] Alizadeh A., Dyck S.M., Karimi-Abdolrezaee S. (2019). Front. Neurol..

[bib2] Anwar M.A., Al Shehabi T.S., Eid A.H. (2016). Front. Cell. Neurosci..

[bib3] Assarsson E., Lundberg M., Holmquist G., Björkesten J., Thorsen S.B., Ekman D., Eriksson A., Dickens E.R., Ohlsson S., Edfeldt G., Andersson A.C., Lindstedt P., Stenvang J., Gullberg M., Fredriksson S. (2014). PLoS One.

[bib4] Capirossi R., Piunti B., Fernández M., Maietti E., Rucci P., Negrini S., Giovannini T., Kiekens C., Calzà L. (2020). Int. J. Mol. Sci..

[bib5] Dalkilic T., Fallah N., Noonan V.K., Salimi Elizei S., Dong K., Belanger L., Ritchie L., Tsang A., Bourassa-Moreau E., Heran M.K.S., Paquette S.J., Ailon T., Dea N., Street J., Fisher C.G., Dvorak M.F., Kwon B.K. (2018). J. Neurotrauma.

[bib6] Dyhrfort P., Shen Q., Clausen F., Thulin M., Enblad P., Kamali-Moghaddam M., Lewén A., Hillered L. (2019). J. Neurotrauma.

[bib7] Fernández M., Baldassarro V.A., Capirossi R., Montevecchi R., Bonavita J., Cescatti M., Giovannini T., Giovannini G., Uneddu M., Giovanni G., Giardino L., Calzà L. (2020). J. Neurotrauma.

[bib8] Filiano A.J., Gadani S.P., Kipnis J. (2017). Nat. Rev. Neurosci..

[bib9] Fleming J.C., Norenberg M.D., Ramsay D.A., Dekaban G.A., Marcillo A.E., Saenz A.D., Pasquale-Styles M., Dietrich W.D., Weaver L.C. (2006). Brain.

[bib10] Hachem L.D., Fehlings M.G. (2021). Neurosurg. Clin..

[bib11] Huang W., Vodovotz Y., Kusturiss M.B., Barclay D., Greenwald K., Boninger M.L., Coen P.M., Brienza D., Sowa G. (2014). Pharm. Manag. PM R.

[bib12] Jin L.-Y., Li J., Wang K.-F., Xia W.-W., Zhu Z.-Q., Wang C.-R., Li X.-F., Liu H.-Y. (2021). J. Neurotrauma.

[bib13] Kuhle J., Gaiottino J., Leppert D., Petzold A., Bestwick J.P., Malaspina A., Lu C.H., Dobson R., Disanto G., Norgren N., Nissim A., Kappos L., Hurlbert J., Yong V.W., Giovannoni G., Casha S. (2015). J. Neurol. Neurosurg. Psychiatry.

[bib14] Kwon B.K., Stammers A.M.T., Belanger L.M., Bernardo A., Chan D., Bishop C.M., Slobogean G.P., Zhang H., Umedaly H., Giffin M., Street J., Boyd M.C., Paquette S.J., Fisher C.G., Dvorak M.F. (2010). J. Neurotrauma.

[bib15] Kwon B.K., Streijger F., Fallah N., Noonan V.K., Bélanger L.M., Ritchie L., Paquette S.J., Ailon T., Boyd M.C., Street J., Fisher C.G., Dvorak M.F. (2017). J. Neurotrauma.

[bib16] Liu X., Zhang Y., Wang Y., Qian T. (2021). World Neurosurg.

[bib17] Lundberg M., Eriksson A., Tran B., Assarsson E., Fredriksson S. (2011). Nucleic Acids Res..

[bib18] Mogensen F.L.H., Delle C., Nedergaard M. (2021). Int. J. Mol. Sci..

[bib19] Pouw M.H., Kwon B.K., Verbeek M.M., Vos P.E., Van Kampen A., Fisher C.G., Street J., Paquette S.J., Dvorak M.F., Boyd M.C., Hosman A.J.F., Van De Meent H. (2014). Spinal Cord.

[bib20] Roberts T.T., Leonard G.R., Cepela D.J. (2017). Clin. Orthop. Relat. Res..

[bib21] Schwab J.M., Zhang Y., Kopp M.A., Brommer B., Popovich P.G. (2014). Exp. Neurol..

[bib22] Sengupta M.B., Basu M., Iswarari S., Mukhopadhyay K.K., Sardar K.P., Acharyya B., Mohanty P.K., Mukhopadhyay D. (2014). PLoS One.

[bib23] Skinnider M.A., Rogalski J., Tigchelaar S., Manouchehri N., Prudova A., Jackson A.M., Nielsen K., Jeong J., Chaudhary S., Shortt K., Gallagher-Kurtzke Y., So K., Fong A., Gupta R., Okon E.B., Rizzuto M.A., Dong K., Streijger F., Belanger L., Ritchie L., Tsang A., Christie S., Mac-Thiong J.M., Bailey C., Ailon T., Charest-Morin R., Dea N., Wilson J.R., Dhall S., Paquette S., Street J., Fisher C.G., Dvorak M.F., Shannon C., Borchers C., Balshaw R., Foster L.J., Kwon B.K. (2021). Mol. Cell. Proteomics.

[bib24] Streijger F., Skinnider M.A., Rogalski J.C., Balshaw R., Shannon C.P., Prudova A., Belanger L., Ritchie L., Tsang A., Christie S., Parent S., Mac-Thiong J.M., Bailey C., Urquhart J., Ailon T., Paquette S., Boyd M., Street J., Fisher C.G., Dvorak M.F., Borchers C.H., Foster L.J., Kwon B.K. (2017). J. Neurotrauma.

[bib25] Yang L., Blumbergs P.C., Jones N.R., Manavis J., Sarvestani G.T., Ghabriel M.N. (2004). Spine.

[bib26] Zrzavy T., Schwaiger C., Wimmer I., Berger T., Bauer J., Butovsky O., Schwab J.M., Lassmann H., Höftberger R. (2021). Brain.

