# Supplementary material for: Cerebrospinal fluid and peripheral blood proteomics in Traumatic Spinal Cord Injury: A prospective pilot study
Source: Brain Spine. 2022 Jun 15;2:100906. doi: 10.1016/j.bas.2022.100906 (PMC9560581; doi:10.1016/j.bas.2022.100906)
Supplement: Multimedia component 1 [file mmc1.docx]

| Table S1 Time from sample collection to processing | | |
| --- | --- | --- |
|  | Peripheral blood | Cerebrospinal fluid |
| Day 0 | 37 min (10-73 min) | 25 min (15-43 min) |
| Day 9 | 31 min (14-51 min) | 30 min (18-66 min) |
| Day 148 | 68 min (24-115 min) | 50 min (15-84 min) |
| Non-TSCI* | 43 min (30-49 min) | 12 min (9-18 min) |
| *Non-TSCI refers to the reference group of patients without spinal cord injury. | | |
