# Supplementary material for: Cerebrospinal fluid and peripheral blood proteomics in Traumatic Spinal Cord Injury: A prospective pilot study
Source: Brain Spine. 2022 Jun 15;2:100906. doi: 10.1016/j.bas.2022.100906 (PMC9560581; doi:10.1016/j.bas.2022.100906)
Supplement: Multimedia component 2 [file mmc2.docx]

| Table 2 Comparison of protein expression between non-traumatic spinal cord injury (TSCI) patients and TSCI patients | | | | | | | | | | | | | | | | | | | |
| --- | --- | --- | --- | --- | --- | --- | --- | --- | --- | --- | --- | --- | --- | --- | --- | --- | --- | --- | --- |
|  | Peripheral blood | | | | | | | | |  | Cerebrospinal fluid | | | | | | | | |
|  | Day 0 vs non-TSCI | | | Day 9 vs non-TSCI | | | Day 148 vs non-TSCI | | |  | Day 0 vs non-TSCI | | | Day 9 vs non-TSCI | | | Day 148 vs non-TSCI | | |
| Proteins | Log_2_FC | P value | P_Adj_ value | Log_2_FC | P value | P_Adj_ value | Log_2_FC | P value | P_Adj_ value |  | Log_2_FC | P value | P_Adj_ value | Log_2_FC | P value | P_Adj_ value | Log_2_FC | P value | P_Adj_ value |
| EN-RAGE | 1.08 | 8.3E-06* | 1.3E-04* | 2.87 | 8.6E-07* | 1.6E-05* | 1.09 | 6.6E-04* | 6.0E-03* |  | 1.30 | 2.8E-02* | 9.3E-02 | 1.40 | 2.9E-02* | 9.6E-02 | -0.17^§^ | 4.3E-01^§^ | 6.0E-01^§^ |
| TRANCE | -1.23 | 1.5E-05* | 2.3E-04* | -0.93 | 8.2E-04* | 7.1E-03* | 0.36 | 1.8E-01 | 3.5E-01 |  | n/a | n/a | n/a | n/a | n/a | n/a | n/a | n/a | n/a |
| Flt3L | -0.84 | 3.4E-05* | 5.0E-04* | 0.01 | 9.6E-01 | 9.8E-01 | 0.18 | 2.2E-01 | 3.9E-01 |  | 0.45 | 1.8E-02* | 6.7E-02 | 0.57 | 3.3E-02* | 1.0E-01 | 0.36 | 1.3E-01 | 2.8E-01 |
| IL-6 | 2.59 | 5.8E-05* | 7.9E-04* | 1.65 | 1.2E-03* | 8.6E-03* | -0.34 | 4.9E-01 | 6.5E-01 |  | 6.28 | 1.1E-06* | 2.1E-05* | 3.13 | 1.7E-04* | 1.8E-03* | 0.38 | 5.3E-02 | 1.4E-01 |
| IL-10 | 1.95 | 1.3E-04* | 1.5E-03* | 0.96 | 7.7E-05* | 9.6E-04* | 0.44 | 5.4E-02 | 1.4E-01 |  | 2.19 | 3.6E-06* | 6.1E-05* | n/a | n/a | n/a | n/a | n/a | n/a |
| CCL23 | 0.69 | 6.9E-04* | 6.1E-03* | 0.53 | 5.1E-02 | 1.4E-01 | 0.14 | 5.0E-01 | 6.6E-01 |  | 1.14 | 9.5E-05* | 1.1E-03* | 1.60 | 1.2E-04* | 1.4E-03* | 1.05 | 6.6E-04* | 6.0E-03* |
| MCP-3 | 0.89 | 8.7E-04* | 7.3E-03* | 1.24 | 1.9E-04* | 1.9E-03* | 0.47 | 4.1E-02* | 1.2E-01 |  | 2.66 | 7.8E-05* | 9.7E-04* | 2.00 | 3.1E-06* | 5.3E-05* | 0.20^§^ | 1.1E-01^§^ | 2.5E-01^§^ |
| IL-8 | 1.29 | 9.8E-04* | 7.3E-03* | 1.44 | 5.8E-03* | 2.9E-02* | 0.64 | 2.5E-02* | 8.8E-02 |  | 4.29 | 1.7E-07* | 3.4E-06* | 1.61 | 1.7E-04* | 1.8E-03* | 0.71 | 4.2E-02* | 1.2E-01 |
| IL-20RA | 0.28 | 1.4E-03* | 9.8E-03* | 0.39 | 3.7E-03* | 2.1E-02* | 0.24 | 1.1E-01 | 2.4E-01 |  | -0.03 | 6.4E-01 | 7.5E-01 | -0.26 | 2.6E-03* | 1.6E-02* | -0.14 | 9.9E-02 | 2.2E-01 |
| TRAIL | -0.46 | 1.5E-03* | 1.0E-02* | -0.69 | 3.2E-01 | 5.0E-01 | 0.17 | 1.4E-01 | 2.9E-01 |  | 0.27 | 2.1E-01 | 3.8E-01 | n/a | n/a | n/a | n/a | n/a | n/a |
| TNFB | -0.59 | 3.0E-03* | 1.7E-02* | 0.07 | 6.9E-01 | 7.9E-01 | 0.17 | 3.9E-01 | 5.6E-01 |  | -0.12 | 4.7E-01 | 6.3E-01 | n/a | n/a | n/a | n/a | n/a | n/a |
| IL-10RB | -0.40 | 4.9E-03* | 2.5E-02* | 0.31 | 6.0E-02 | 1.6E-01 | 0.05 | 6.7E-01 | 7.7E-01 |  | 0.28 | 1.7E-01 | 3.2E-01 | 0.93 | 2.2E-03* | 1.4E-02* | 0.40 | 8.3E-02 | 2.0E-01 |
| CCL25 | -0.80 | 5.8E-03* | 2.9E-02* | -0.25 | 2.9E-01 | 4.7E-01 | 0.15 | 5.4E-01 | 6.9E-01 |  | 0.51 | 5.8E-02 | 1.5E-01 | 0.54 | 1.7E-01 | 3.2E-01 | 0.56 | 7.2E-02 | 1.8E-01 |
| SCF | -0.43 | 6.6E-03* | 3.2E-02* | -1.11 | 5.1E-04* | 4.8E-03* | -0.31 | 1.8E-01 | 3.4E-01 |  | 0.38 | 1.1E-01 | 2.5E-01 | 0.63 | 1.5E-01 | 3.0E-01 | 0.26 | 3.1E-01 | 4.9E-01 |
| TWEAK | -0.48 | 1.1E-02* | 4.6E-02* | -0.76 | 1.1E-04* | 1.3E-03* | -0.04 | 7.1E-01 | 8.0E-01 |  | -0.79 | 9.9E-04* | 7.3E-03* | n/a | n/a | n/a | n/a | n/a | n/a |
| CCL11 | -0.55 | 1.1E-02* | 4.6E-02* | -0.31 | 4.4E-02* | 1.3E-01 | 0.28 | 1.1E-01 | 2.4E-01 |  | 0.77 | 5.2E-03* | 2.7E-02* | 0.39 | 9.4E-01 | 9.5E-01 | 0.34 | 8.9E-03* | 4.0E-02* |
| IL-24 | 0.49 | 1.1E-02* | 4.6E-02* | 0.54 | 1.1E-02* | 4.6E-02* | 0.23 | 2.5E-01 | 4.3E-01 |  | n/a | n/a | n/a | n/a | n/a | n/a | n/a | n/a | n/a |
| X4E-BP1 | -0.97 | 1.5E-02* | 5.6E-02 | 0.28 | 3.2E-01 | 5.0E-01 | -0.35 | 4.3E-01 | 6.0E-01 |  | 2.01 | 8.9E-05* | 1.1E-03* | 1.65 | 2.8E-03* | 1.7E-02* | 0.58 | 7.6E-02 | 1.8E-01 |
| LIF | 0.43 | 1.7E-02* | 6.3E-02 | 0.61 | 3.7E-03* | 2.1E-02* | 0.24 | 9.7E-02 | 2.2E-01 |  | 5.46 | 1.7E-07* | 3.4E-06* | 2.23 | 8.7E-05* | 1.1E-03* | 0.44 | 2.1E-02* | 7.5E-02 |
| CCL4 | 0.59 | 1.9E-02* | 6.8E-02 | 0.83 | 2.5E-03* | 1.5E-02* | 0.24 | 5.4E-01 | 6.8E-01 |  | 2.17 | 4.7E-05* | 6.4E-04* | 0.57 | 5.1E-02 | 1.4E-01 | 0.48 | 2.4E-02* | 8.4E-02 |
| FGF-21 | 1.30 | 2.7E-02* | 9.1E-02 | -1.69 | 3.2E-03* | 1.8E-02* | -0.60 | 3.0E-01 | 4.8E-01 |  | 1.32 | 4.5E-03* | 2.4E-02* | 0.14 | 6.1E-01 | 7.4E-01 | 0.45 | 2.4E-01 | 4.2E-01 |
| uPA | -0.32 | 3.0E-02* | 9.8E-02 | 0.08 | 4.8E-01 | 6.3E-01 | -0.03 | 7.9E-01 | 8.6E-01 |  | 0.18 | 4.8E-01 | 6.4E-01 | 0.38 | 8.9E-01 | 9.3E-01 | -0.03 | 8.9E-01 | 9.3E-01 |
| OSM | -0.67 | 3.3E-02* | 1.0E-01 | 0.44 | 6.1E-01 | 7.4E-01 | -0.78 | 4.1E-02* | 1.2E-01 |  | 3.48 | 1.8E-06* | 3.3E-05* | 1.54 | 3.4E-03* | 1.9E-02* | 0.38 | 6.5E-03* | 3.2E-02* |
| CSF-1 | 0.20 | 4.0E-02* | 1.2E-01 | 0.57 | 2.1E-06* | 3.6E-05* | 0.19 | 4.1E-02* | 1.2E-01 |  | 0.70 | 1.7E-03* | 1.1E-02* | 0.22 | 2.2E-01 | 3.9E-01 | 0.03 | 8.0E-01 | 8.7E-01 |
| FGF-19 | 0.63 | 4.3E-02* | 1.2E-01 | 2.03 | 1.9E-06* | 3.4E-05* | 1.80 | 6.5E-05* | 8.5E-05* |  | 0.28 | 1.6E-01 | 3.1E-01 | -0.24 | 1.1E-01 | 2.3E-01 | 0.01 | 9.4E-01 | 9.5E-01 |
| CCL3 | 0.52 | 5.5E-02 | 1.5E-01 | 1.17 | 1.1E-03* | 7.7E-03* | 0.63 | 6.0E-02 | 1.6E-01 |  | 2.16 | 1.4E-04* | 1.5E-03* | 1.02 | 5.9E-03* | 2.9E-02* | 1.23 | 2.3E-04* | 2.3E-03* |
| DNER | -0.23 | 7.4E-02 | 1.8E-01 | -0.51 | 8.0E-03* | 3.7E-02* | -0.27 | 8.3E-02 | 2.0E-01 |  | -0.07 | 1.7E-01 | 3.2E-01 | -0.13 | 1.1E-01 | 2.3E-01 | -0.11 | 7.7E-02 | 1.9E-01 |
| CCL20 | 0.83 | 9.8E-02 | 2.2E-01 | 1.00 | 1.6E-02* | 6.1E-02 | 0.23 | 3.9E-01 | 5.6E-01 |  | 4.05 | 7.5E-05* | 9.5E-04* | 1.30 | 1.5E-01 | 3.0E-01 | 0.18 | 4.4E-01 | 6.1E-01 |
| IL-20 | 0.31 | 9.8E-02 | 2.2E-01 | 0.20 | 6.8E-02 | 1.7E-01 | 0.04^§^ | 6.7E-01^§^ | 7.7E-01^§^ |  | 0.17^§^ | 1.2E-02^§^ | 4.9E-02^§^ | 0.06^§^ | 2.9E-01^§^ | 4.7E-01^§^ | -0.06^§^ | 3.6E-01^§^ | 5.4E-01^§^ |
| VEGF-A | 0.41 | 1.2E-01 | 2.5E-01 | 0.87 | 7.7E-03* | 3.6E-02* | 0.42 | 1.0E-01 | 2.3E-01 |  | 0.15 | 4.6E-01 | 6.3E-01 | -0.02 | 2.2E-01 | 3.9E-01 | -0.29 | 1.6E-01 | 3.1E-01 |
| NRTN | 0.09^§^ | 1.3E-01^§^ | 2.6E-01^§^ | 0.13 | 1.3E-01 | 2.8E-01 | 0.01^§^ | 5.7E-01^§^ | 7.1E-01^§^ |  | n/a | n/a | n/a | n/a | n/a | n/a | n/a | n/a | n/a |
| STAMBP | -0.30 | 1.4E-01 | 2.8E-01 | 0.06 | 9.3E-01 | 9.5E-01 | 0.20 | 7.5E-01 | 8.3E-01 |  | 1.52 | 2.8E-05* | 4.1E-04* | 0.67 | 9.0E-03* | 4.0E-02* | 0.03 | 8.0E-01 | 8.7E-01 |
| IL-10RA | -0.13 | 1.4E-01 | 2.8E-01 | 0.09 | 8.0E-01 | 8.6E-01 | 0.13 | 9.6E-01 | 9.7E-01 |  | 0.09 | 1.3E-01 | 2.6E-01 | 0.17^§^ | 4.6E-02^§^ | 1.3E-01^§^ | 0.03^§^ | 6.6E-01^§^ | 7.7E-01^§^ |
| SIRT2 | -0.50 | 1.5E-01 | 3.0E-01 | 0.28 | 7.0E-01 | 7.9E-01 | 0.17 | 7.8E-01 | 8.5E-01 |  | 0.67 | 7.7E-02 | 1.9E-01 | 0.34 | 2.7E-01 | 4.5E-01 | -0.14 | 5.6E-01 | 7.1E-01 |
| IL-2 | 0.17^§^ | 1.5E-01^§^ | 3.0E-01^§^ | 0.26 | 3.0E-03* | 1.7E-02* | -0.001^§^ | 9.2E-01^§^ | 9.5E-01^§^ |  | n/a | n/a | n/a | n/a | n/a | n/a | n/a | n/a | n/a |
| IL-12B | -0.29 | 1.6E-01 | 3.1E-01 | 0.59 | 2.6E-02* | 8.9E-02 | 0.46 | 9.5E-02 | 2.2E-01 |  | 0.05 | 7.1E-01 | 8.0E-01 | 0.27 | 5,4E-01 | 6.8E-01 | 0.24 | 2.7E-01 | 4.5E-01 |
| NT-3 | -0.11 | 1.8E-01 | 3.4E-01 | -0.27 | 8.2E-03* | 3.7E-02* | 0.13 | 9.6E-01 | 9.7E-01 |  | -0.08^§^ | 4.4E-01^§^ | 6.0E-01^§^ | 0.02^§^ | 8.0E-01^§^ | 8.6E-01^§^ | -0.05^§^ | 5.4E-01^§^ | 6.9E-01^§^ |
| MMP-1 | 0.51 | 1.9E-01 | 3.5E-01 | 1.40 | 1.6E-03* | 1.1E-02* | 1.00 | 1.6E-02* | 6.1E-02 |  | 2.12 | 5.1E-04* | 4.8E-03* | 0.96 | 9.8E-01 | 9.8E-01 | -0.53 | 9.5E-02 | 2.2E-01 |
| FGF-5 | -0.08 | 2.0E-01 | 3.7E-01 | -0.001 | 5.7E-01 | 7.1E-01 | 0.04 | 7.2E-01 | 8.1E-01 |  | -0.23 | 2.4E-01 | 4.1E-01 | -0.20 | 4.1E-01 | 5.8E-01 | -0.58 | 3.7E-02 | 1.1E-01 |
| SLAMF-1 | -0.23 | 2.0E-01 | 3.7E-01 | 0.06 | 9.2E-01 | 9.5E-01 | -0.17 | 3.6E-01 | 5.4E-01 |  | n/a | n/a | n/a | n/a | n/a | n/a | n/a | n/a | n/a |
| CD40 | -0.02 | 2.2E-01 | 3.9E-01 | 0.55 | 9.8E-02 | 2.2E-01 | 0.40 | 1.6E-01 | 3.1E-01 |  | 0.34 | 1.0E-01 | 2.3E-01 | 0.72 | 4.4E-02* | 1.3E-01 | 0.32 | 1.7E-01 | 3.2E-01 |
| CD244 | 0.02 | 2.2E-01 | 3.9E-01 | 0.14 | 5.9E-01 | 7.2E-01 | 0.37 | 3.6E-01 | 5.4E-01 |  | 0.46 | 1.1E-02* | 4.5E-02* | 0.37 | 1.7E-01 | 3.2E-01 | 0.41 | 5.6E-02 | 1.5E-01 |
| TNFSF14 | 0.55 | 2.3E-01 | 4.1E-01 | 1.42 | 1.2E-03* | 8.9E-03* | 0.21 | 4.8E-01 | 6.4E-01 |  | 0.24 | 1.8E-01 | 3.3E-01 | 0.15 | 4.2E-01 | 5.9E-01 | 0.28 | 1.1E-01 | 2.3E-01 |
| TSLP | -0.19^§^ | 2.4E-01^§^ | 4.1E-01^§^ | -0.10^§^ | 5.4E-01^§^ | 6.9E-01^§^ | -0.15^§^ | 5.2E-01^§^ | 6.7E-01^§^ |  | 0.75 | 9.6E-04* | 7.3E-03* | 0.62^§^ | 3.3E-02^§^ | 1.0E-01^§^ | 0.26^§^ | 1.2E-01^§^ | 2.6E-01^§^ |
| CXCL5 | -0.65 | 2.5E-01 | 4.2E-01 | 0.30 | 6.6E-02 | 1.7E-01 | -0.77 | 2.5E-01 | 4.2E-01 |  | 1.33 | 1.2E-02* | 4.8E-02* | 1.10 | 2.5E-02* | 8.5E-02 | 0.05 | 6.5E-01 | 7.6E-01 |
| MCP-1 | 0.26 | 2.5E-01 | 4.2E-01 | 0.30 | 6.6E-02 | 1.7E-01 | 0.32 | 6.8E-02 | 1.7E-01 |  | 2.02 | 6.2E-07* | 1.2E-05* | 0.98 | 5.2E-04* | 4.9E-03* | 1.09 | 3-0E-04* | 3.0E-03* |
| MCP-4 | -0.06 | 2.7E-01 | 4.5E-01 | 0.23 | 6.3E-01 | 7.5E-01 | 0.40 | 7.6E-01 | 8.4E-01 |  | 1.03 | 3.3E-03* | 1.9E-02* | 1.53 | 7.5E-03* | 3.5E-02* | 0.34 | 2.0E-01 | 3.6E-01 |
| LAP TGF-beta | 0.16 | 2.7E-01 | 4.5E-01 | 0.45 | 7.6E-01 | 8.4E-01 | 0.26 | 3.3E-01 | 5.1E-01 |  | 0.45 | 5.2E-02 | 1.4E-01 | 0.71 | 3.8E-02* | 1.2E-01 | 0.18 | 4.0E-01 | 5.8E-01 |
| TNFRSF9 | -0.21 | 2.7E-01 | 4.5E-01 | 0.11 | 5.6E-01 | 7.0E-01 | 0.32 | 1.6E-01 | 3.1E-01 |  | 0.66 | 7.6E-03* | 3.5E-02* | 0.82 | 1.1E-02* | 4.6E-02* | 0.50 | 9.2E-02 | 2.1E-01 |
| ST1A1 | 0.41 | 2.8E-01 | 4.6E-01 | 0.25 | 6.6E-01 | 7.7E-01 | 0.39 | 3.2E-01 | 5.0E-01 |  | 0.93 | 4.5E-03* | 2.4E-02* | 0.37^§^ | 1.6E-01^§^ | 3.1E-01^§^ | 0.09^§^ | 4.7E-01^§^ | 6.3E-01^§^ |
| AXIN1 | 0.25 | 3.2E-01 | 5.0E-01 | 0.66 | 3.5E-01 | 5.3E-01 | 0.80 | 1.7E-01 | 3.2E-01 |  | 0.63 | 8.8E-04* | 7.3E-03* | 0.24^§^ | 2.2E-01^§^ | 3.9E-01^§^ | -0.05^§^ | 5.4E-01^§^ | 6.8E-01^§^ |
|  |  |  |  |  |  |  |  |  |  |  |  |  |  |  |  |  |  |  |  |
| IL-33 | 0.12^§^ | 3.4E-01^§^ | 5.2E-01^§^ | 0.16^§^ | 2.3E-01^§^ | 4.0E-01^§^ | -0.19^§^ | 1.1E-01^§^ | 2.3E-01^§^ |  | 0.33^§^ | 3.8E-02^§^ | 1.2E-01^§^ | 0.11^§^ | 3.4E-01^§^ | 5.2E-01^§^ | 0.10^§^ | 3.4E-01^§^ | 5.2E-01^§^ |
| CD5 | -0.06 | 3.7E-01 | 5.4E-01 | 0.09 | 9.6E-01 | 9.7E-01 | 0.28 | 3.6E-01 | 5.4E-01 |  | 0.62 | 2.1E-02* | 7.5E-02 | 0.61 | 9.7E-02 | 2.2E-01 | 0.50 | 4.3E-02* | 1.2E-01 |
| TGF-alpha | 0.071 | 3.7E-01 | 5.4E-01 | 0.32 | 1.6E-01 | 3.2E-01 | 0.08 | 4.6E-01 | 6.3E-01 |  | -0.20 | 2.0E-01 | 3.7E-01 | -0.17 | 4.3E-01 | 6.0E-01 | -0.04 | 7.9E-01 | 8.6E-01 |
| CD8A | -0.30 | 3.9E-01 | 5.7E-01 | -0.05 | 8.4E-01 | 8.9E-01 | 0.35 | 8.2E-02 | 1.9E-01 |  | 0.25 | 2.6E-01 | 4.3E-01 | 0.45 | 2.9E-02* | 9.6E-02 | 0.38 | 1.8E-01 | 3.4E-01 |
| CXCL1 | 0.26 | 4.0E-01 | 5.7E-01 | 0.73 | 5.4E-02 | 1.4E-01 | 0.42 | 2.4E-01 | 4.2E-01 |  | 3.93 | 1.8E-05* | 2.7E-04* | 1.20 | 3.2E-02* | 1.0E-01 | 0.33 | 2.0E-01 | 3.7E-01 |
| OPG | 0.14 | 4.0E-01 | 5.7E-01 | 0.29 | 7.3E-02 | 1.8E-01 | -0.11 | 3.9E-01 | 5.6E-01 |  | 0.99 | 1.2E-03* | 8.8E-03* | 0.58 | 7.5E-02 | 1.8E-01 | 0.18 | 4.9E-01 | 6.5E-01 |
| CDCP1 | -0.24 | 4.0E-01 | 5.8E-01 | 0.75 | 1.4E-02 | 5.3E-02 | 0.24 | 3.3E-01 | 5.1E-01 |  | -0.08 | 5.8E-01 | 7.2E-01 | -0.07 | 2.0E-01 | 3.7E-01 | 0.53 | 1.1E-02* | 4.7E-02* |
| CD6 | 0.019 | 4.1E-01 | 5.8E-01 | -0.26 | 1.5E-01 | 3.0E-01 | 0.41 | 2.0E-01 | 3.7E-01 |  | 0.33 | 6.3E-02 | 1.6E-01 | 0.26 | 5.0E-01 | 6.6E-01 | 0.38 | 7.9E-02 | 1.9E-01 |
| CST5 | -0.14 | 4.1E-01 | 5.8E-01 | 0.03 | 8.8E-01 | 9.2E-01 | 0.18 | 3.8E-01 | 5.6E-01 |  | 0.02 | 5.1E-01 | 6.7E-01 | 0.07 | 9.8E-01 | 9.8E-01 | -0.01 | 6.6E-01 | 7.7E-01 |
| IL-17C | 0.63 | 4.1E-01 | 5.8E-01 | 0.63 | 3.6E-02* | 1.1E-01 | 0.27 | 1.1E-01 | 2.4E-01 |  | 0.29^§^ | 3.1E-02^§^ | 1.0E-01^§^ | 0.53^§^ | 5.8E-02^§^ | 1.5E-01^§^ | 0.20^§^ | 1.6E-01^§^ | 3.2E-01^§^ |
| ARTN | -0.08 | 4.5E-01 | 6.2E-01 | 0.12 | 4.5E-01 | 6.2E-01 | 0.01 | 9.0E-01 | 9.3E-01 |  | 0.14 | 1.4E-01 | 2.9E-01 | 0.009^§^ | 9.2E-01^§^ | 9.5E-01^§^ | -0.08^§^ | 3.1E-01^§^ | 4.9E-01^§^ |
| TNF | 0.13 | 4.7E-01 | 6.3E-01 | 0.87 | 1.1E-04* | 1.3E-03* | 0.45 | 8.1E-03* | 3.7E-02* |  | 0.98 | 1.2E-04* | 1.4E-03* | 0.84 | 4.4E-05* | 6.0E-04* | 0.53 | 2.2E-03* | 1.4E-02* |
| IFN-gamma | -0.77 | 4.9E-01 | 6.4E-01 | 0.72 | 2.0E-02* | 7.3E-02 | 0.28 | 3.6E-02* | 1.1E-01 |  | 0.54 | 9.7E-02 | 2.2E-01 | 1.90 | 1.7E-02* | 6.3E-02 | 0.67 | 5.0E-02 | 1.4E-01 |
| FGF-23 | -0.11 | 5.0E-01 | 6.6E-01 | 0.40 | 1.4E-02 | 5.5E-02 | 0.19 | 2.1E-01 | 3.8E-01 |  | -0.16 | 2.8E-01 | 4.5E-01 | 0.24 | 4.3E-01 | 6.0E-01 | 0.07^§^ | 4.7E-01^§^ | 6.3E-01^§^ |
| CXCL6 | 0.29 | 5.4E-01 | 6.8E-01 | 0.43 | 3.6E-01 | 5.4E-01 | 0.55 | 5.4E-01 | 6.8E-01 |  | 2.34 | 2.5E-04* | 2.5E-03* | 1.30 | 1.8E-03* | 1.2E-02* | 0.51 | 5.0E-02 | 1.4E-01 |
| LIF-R | -0.05 | 5.5E-01 | 7.0E-01 | 0.07 | 5.2E-01 | 6.7E-01 | -0.09 | 2.9E-01 | 4.7E-01 |  | -0.17 | 2.7E-01 | 4.5E-01 | -0.03 | 8.4E-01 | 8.9E-01 | -0.33 | 5.8E-02 | 1.5E-01 |
| CXCL9 | -0.20 | 5.8E-01 | 7.2E-01 | 0.34 | 5.1E-01 | 6.6E-01 | 0.55 | 7.6E-02 | 1.8E-01 |  | 1.10 | 2.2E-02* | 7.3E-02 | 1.73 | 2.2E-03* | 1.4E-02* | 1.32 | 5.5E-03* | 2.8E-02* |
| CASP-8 | 0.30 | 5.9E-01 | 7.3E-01 | 0.27 | 7.6E-01 | 8.4E-01 | 0.11 | 9.2E-01 | 9.5E-01 |  | 1.44 | 2.0E-04* | 2.0E-03* | 0.53^§^ | 1.5E-02^§^ | 5.8E-02^§^ | 0.17^§^ | 1.2E-01^§^ | 2.5E-01^§^ |
| GDNF | -0.04 | 6.0E-01 | 7.3E-01 | 0.32 | 4.9E-02* | 1.4E-01 | 0.06 | 5.6E-01 | 7.0E-01 |  | n/a | n/a | n/a | n/a | n/a | n/a | n/a | n/a | n/a |
| IL-18R1 | 0.08 | 6.0E-01 | 7.3E-01 | 0.94 | 4.8E-04* | 4.6E-03* | 0.11 | 5.6E-01 | 7.0E-01 |  | 0.65 | 2.1E-02* | 7.7E-02 | 1.10 | 1.8E-02* | 6.6E-02 | 0.40 | 1.2E-01 | 2.5E-01 |
| ADA | 0.18 | 6.2E-01 | 7.5E-01 | 0.23 | 6.1E-01 | 7.4E-01 | 0.13 | 6.6E-01 | 7.7E-01 |  | -0.08 | 6.7E-01 | 7.7E-01 | 0.07 | 4.7E-01 | 6.3E-01 | -0.43 | 2.9E-02* | 9.6E-02 |
| IL-17A | 0.30 | 6.2E-01 | 7.5E-01 | 1.61 | 1.3E-04* | 1.4E-03* | 0.52 | 1.8E-02* | 6.5E-02 |  | 0.17 | 1.4E-01 | 2.8E-01 | 0.40^§^ | 5.1E-02^§^ | 1.4E-01^§^ | 0.08 | 3.7E-01 | 5.5E-01 |
| CXCL10 | -0.15 | 6.7E-01 | 7.8E.01 | 0.68 | 2.8E-02* | 9.4E-02 | 0.64 | 7.3E-02 | 1.8E-01 |  | 1.25 | 6.1E-04* | 5.6E-03* | 2.04 | 1.1E-04* | 1.3E-03* | 1.32 | 4.5E-03* | 2.4E-02* |
| CCL28 | 0.18 | 6.8E-01 | 7.8E-01 | 0.09 | 5.6E-01 | 7.1E-01 | 0.13 | 4.4E-01 | 6.1E-01 |  | 0.43 | 2.4E-03* | 1.5E-02* | 0.34 | 1.0E-02* | 4.4E-02* | 0.23 | 1.1E-01 | 2.4E-01 |
| IL-15RA | -0.04 | 7.0E-01 | 7.9E-01 | 0.13 | 2.8E-01 | 4.7E-01 | 0.14 | 1.5E-01 | 3.0E-01 |  | 0.11^§^ | 2.6E-01^§^ | 4.4E-01^§^ | 0.22 | 2.7E-01^§^ | 4.5E-01^§^ | 0.13 | 1.0E-01 | 2.3E-01 |
| CCL19 | -0.11 | 7.5E-01 | 8.3E-01 | 0.81 | 3,5E-03* | 2.0E-02* | 0.81 | 2.6E-02* | 9.0E-02 |  | -0.16 | 6.1E-01 | 7.4E-01 | 0.18 | 6.4E-01 | 7.6E-01 | -0.59 | 9.2E-02 | 2.1E-01 |
| IL-5 | -0.42 | 7.7E-01 | 8.5E-01 | -0.42 | 5.7E-01 | 7.1E-01 | -0.66 | 2.2E-01 | 3.9E-01 |  | n/a | n/a | n/a | n/a | n/a | n/a | n/a | n/a | n/a |
| CX3CL1 | 0.04 | 7.8E-01 | 8.5E-01 | -0.02 | 8.8E-01 | 9.2E-01 | -0.04 | 7.6E-01 | 8.3E-01 |  | -0.20 | 2.5E-01 | 4.2E-01 | -0.009 | 9.7E-01 | 9.8E-01 | -0.09 | 6.0E-01 | 7.4E-01 |
| MCP-2 | 0.54 | 8.1E-01 | 8.7E-01 | 0.82 | 1.7E-02* | 6.5E-02 | 0.97 | 1.3E-02* | 5.1E-02 |  | 1.89 | 2.1E-06* | 3.7E-05* | 1.95 | 1.6E-03* | 1.1E-02* | 0.74 | 1.7E-02 | 6.2E-02 |
| IL-22RA1 | 0.15^§^ | 8.1E-01^§^ | 8.7E-01^§^ | 0.35^§^ | 3.6E-01^§^ | 5.4E-01^§^ | 0.13^§^ | 9.2E-01^§^ | 9.5E-01^§^ |  | n/a | n/a | n/a | n/a | n/a | n/a | n/a | n/a | n/a |
| MMP-10 | 0.051 | 8.4E-01 | 8.9E-01 | 0.76 | 7.8E-03* | 3.6E-02* | 0.26 | 3.1E-01 | 4.9E-01 |  | 1.44 | 2.4E-03* | 1.5E-02* | 1.13 | 8.7E-04* | 7.3E-03* | 0.94 | 7.5E-03* | 3.5E-02* |
| HGF | 0.03 | 8.5E-01 | 9.0E-01 | 0.54 | 3.6E-02* | 1.1E-01 | -0.20 | 2.5E-01 | 4.3E-01 |  | 0.28 | 2.3E-01 | 4.1E-01 | 0.29 | 7.3E-01 | 8.1E-01 | -0.09 | 6.6E-01 | 7.7E-01 |
| IL-4 | -0.03^§^ | 8.5E-01^§^ | 9.0E-01^§^ | 0.19 | 3.7E-01 | 5.4E-01 | 0.11^§^ | 5.2E-01^§^ | 6.7E-01^§^ |  | 0.21 | 1.9E-01 | 3.6E-01 | 0.29 | 5.9E-02 | 1.5E-01 | 0.22^§^ | 1.7E-01^§^ | 3.2E-01^§^ |
| CXCL11 | 0.43 | 8.7E-01 | 9.2E-01 | 2.05 | 5.8E-03* | 2.9E-02* | 1.24 | 1.1E-02* | 4.7E-02* |  | 0.96 | 8.0E-03* | 3.7E-02* | 1.72 | 5.4E-03* | 2.8E-02* | 0.92 | 1.8E-02 | 6.6E-02 |
| IL-7 | 0.69 | 9.3E-01 | 9.5E-01 | 0.89 | 1.5E-01 | 3.0E-01 | 0.48 | 1.0E+00 | 1.0E+00 |  | 0.22 | 1.0E-01 | 2.3E-01 | 0.53 | 4.2E-03* | 2.3E-02* | 0.06 | 3.6E-01 | 5.4E-01 |
| IL-18 | -0.01 | 9.8E-01 | 9.8E-01 | 0.28 | 3.2E-01 | 5.0E-01 | 0.21 | 2.7E-01 | 4.5E-01 |  | 1.91 | 4.0E-06* | 6.6E-05* | 1.10 | 6.9E-03* | 3.3E-02* | 0.82 | 6.5E-03* | 3.2E-02* |
| PD-L1 | 0.18 | 1.0E+00 | 1.0E+00 | 0.86 | 1.8E-02* | 6.5E-02 | 0.45 | 8.0E-02 | 1.9E-01 |  | 0.001 | 9.9E-01 | 1.0E+00 | -0.02 | 9.3E-01 | 9.5E-01 | -0.08 | 7.2E-01 | 8.1E-01 |
| IL-1 alpha | n/a | n/a | n/a | n/a | n/a | n/a | n/a | n/a | n/a |  | 0.82 | 7.8E-04* | 6.8E-03* | 4.50^§^ | 1.4E-03^§^ | 9.8E-03^§^ | 0.48^§^ | 8.3E-03^§^ | 3.8E-02^§^ |
| Log_2_ fold change (log_2_FC) and p values (unadjusted and adjusted using the Benjamini-Hochberg method) for the comparison of non-TSCI patients and TSCI patients. A positive log_2_FC value indicates upregulated protein expression in TSCI patients, whereas a negative log_2_FC value indicates downregulated protein expression. *Statistical significance was set at p<0.05. ^§^As medians of both groups tested are below lower limit of detection, caution should be taken as there is higher technical variation of measurements below lower limit of detection. Some proteins were omitted from analysis as >90% of the measurements were below lower limit of detection. These er marked by n/a.  Non-TSCI refers to the reference group of patients without spinal cord injury. | | | | | | | | | | | | | | | | | | | |
