# Supplementary figures and images for: Cerebrospinal fluid and peripheral blood proteomics in Traumatic Spinal Cord Injury: A prospective pilot study
Source: Brain Spine. 2022 Jun 15;2:100906. doi: 10.1016/j.bas.2022.100906 (PMC9560581; doi:10.1016/j.bas.2022.100906)

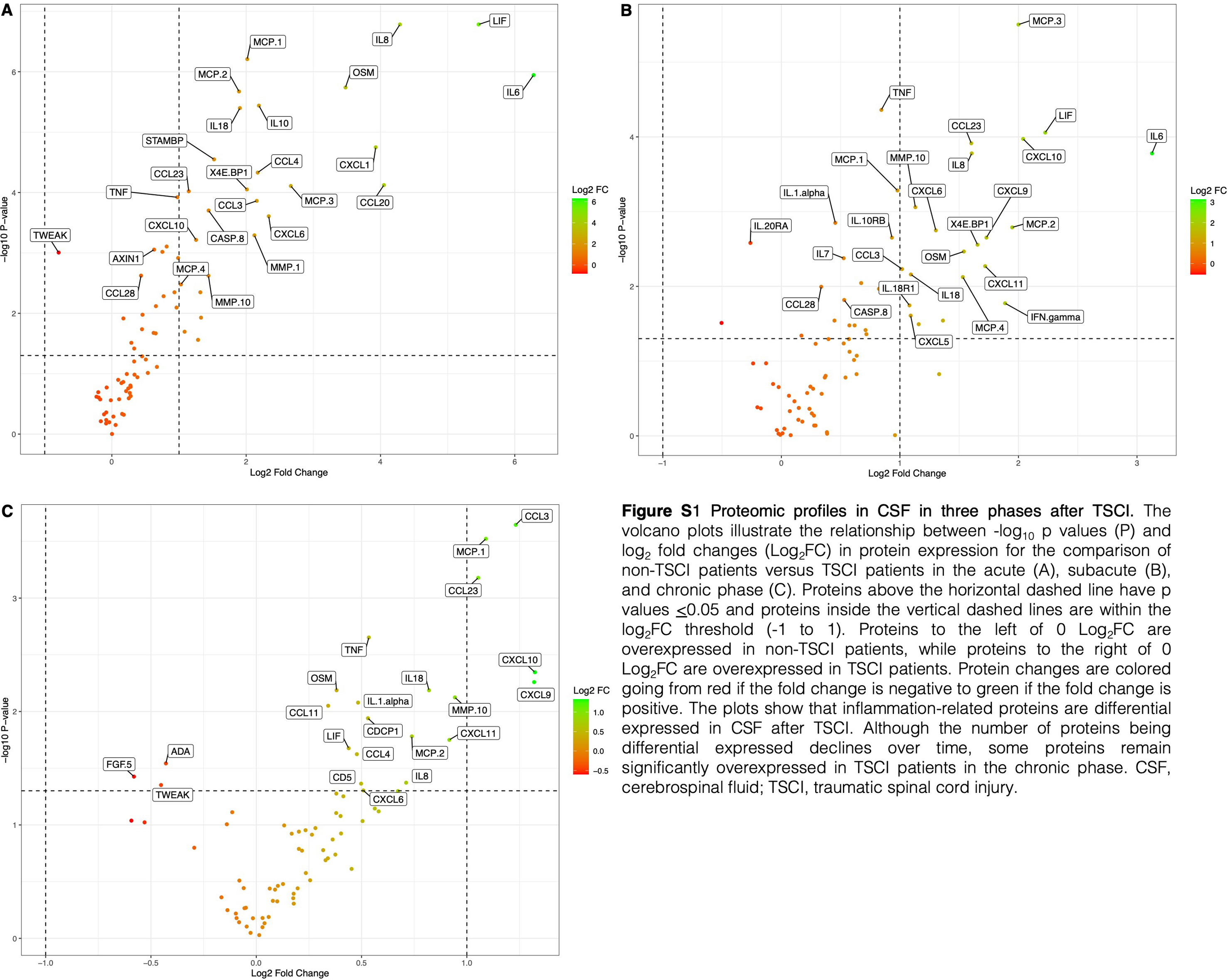

Supplement: Fig. S1 [file figs1.jpg]

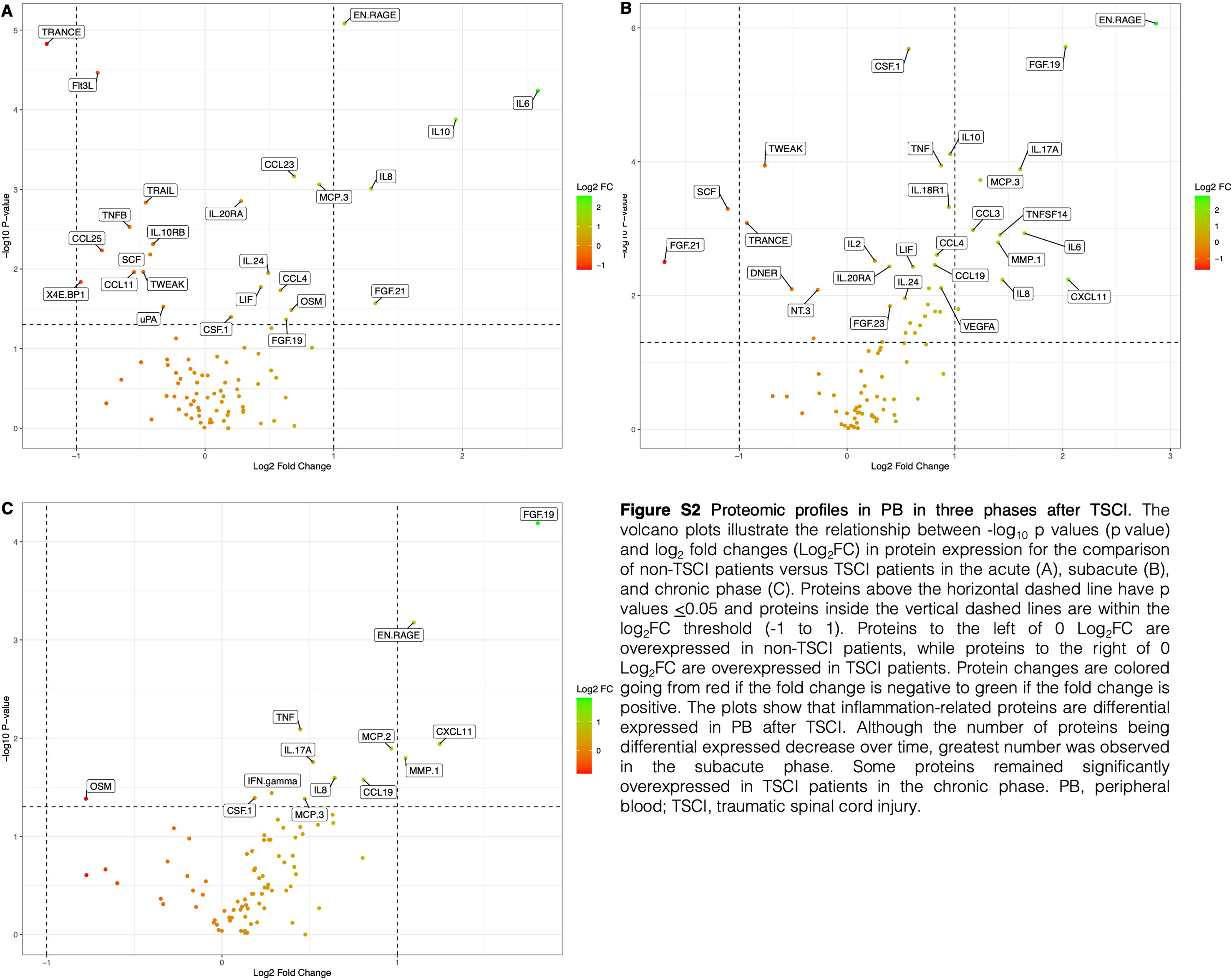

Supplement: Fig. S2 [file figs2.jpg]
